# Slow continuous activity in the circuit of PV-gap reentry successfully diagnosed by an omnipolar technology

**DOI:** 10.1016/j.ipej.2022.05.003

**Published:** 2022-06-02

**Authors:** Masateru Takigawa, Masahiko Goya, Takashi Ikenouchi, Ryusuke Awane, Shinsuke Miyazaki, Tetsuo Sasano

**Affiliations:** Tokyo Medical and Dental University, Tokyo Ika Shika Daigaku, Tokyo, Japan

**Keywords:** Omnipolar, Bipolar, Atrial tachycardia, Mapping, Catheter ablation, AT, atrial tachycardia, AF, atrial fibrillation, CL, cycle length, EGM, electrogram, LA, left atrium, LAA, left atrial appendage, LIPV, left inferior pulmonary vein, LSPV, left superior pulmonary vein, PV, pulmonary vein, PVI, pulmonary vein isolation, RIPV, right inferior pulmonary vein, RSPV, right superior pulmonary vein, RF, radiofrequency

## Case report

The angle between the direction of the bipolar electrodes and wavefront activation direction dramatically affects bipolar electrograms (EGMs), which sometimes affects the activation map.

We present a 56-year-old male under hemodialysis due to nephrosclerosis, who came in for radiofrequency (RF)-ablation of paroxysmal atrial fibrillation (AF). Pulmonary vein isolation (PVI) was achieved on the right side and during PVI on the left side, atrial tachycardia (AT) with cycle length (CL) of 272 ms spontaneously occurred.

**Fig. 1:**
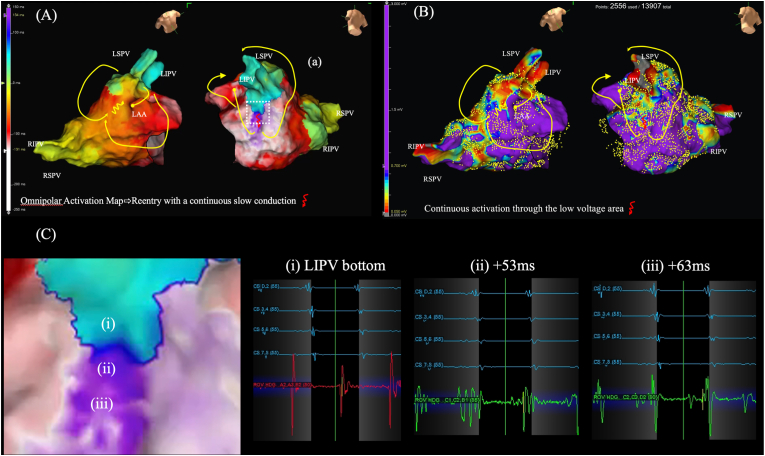
Novel omnipolar activation map (A) and voltage map (B) during tachycardia. Note that slow continuous activation at the bottom of the inferior LIPV was clearly displayed in the low voltage area (red arrow). Magnification of the area of interest (white dotted square) and automatically acquired electrograms in this region (C) demonstrated that continuous activation was displayed in a sequence of (i)→(ii)→(iii), suggesting reentrant activity.

The omnipolar activation map created during tachycardia (2556 used points out of 13907 acquired points) demonstrated the circuit of PV-gap reentrant AT with continuous slow conduction at the bottom of the inferior left PV at the low voltage area (0.12mV) (Fig. 1, Video 1). Entrainment pacing demonstrated that both the entrance of the circuit located on the roof of the left superior pulmonary vein (LSPV) and the exit of the circuit located at the bottom of the left inferior pulmonary vein (LIPV) were inside the circuit of PV-gap reentrant AT. The AT was immediately terminated by RF-application at the bottom of LIPV, followed by ablation to close the gap at the roof of the LSPV. Neither PV-reconduction nor induction of any other ATs were observed under adenosine infusion and isoproterenol drip infusion.

**Fig. 2:**
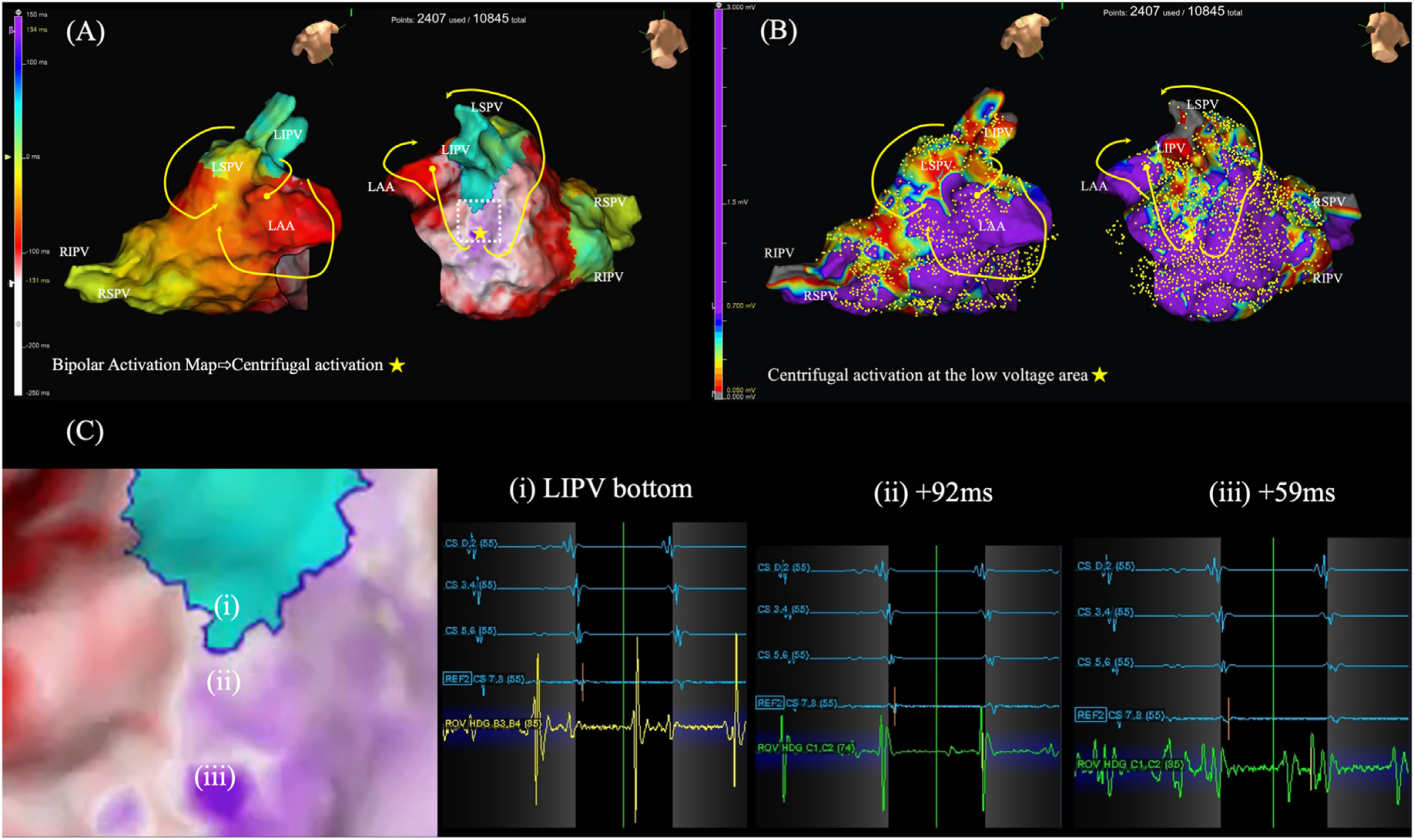
Conventional bipolar activation map (A) and voltage map (B) during tachycardia. Note that slow continuous activation at the bottom of the inferior LIPV was not observed, and instead, centrifugal activation from the lateral LA (yellow star) was observed. Magnification of the area of interest (white dotted square) and automatically acquired electrograms in this region (C) demonstrated that discrete activation was displayed in a sequence of (iii)→(ii)→→→(i). Suggesting focal activity.

Interestingly, when this AT was displayed by a conventional bipolar map (2407 used points out of 10845 acquired points), the AT was mis-interpreted as centrifugal activation at the lateral LA (Fig. 2, Video 2).

Supplementary video related to this article can be found at https://doi.org/10.1016/j.ipej.2022.05.003

The following are the supplementary data related to this article:

Multimedia component 1 1 Multimedia component 1

Multimedia component 2 2 Multimedia component 2

The superiority of novel omnipolar mapping to conventional bipolar mapping in automatically diagnosing the AT circuit was clearly demonstrated in this case.

Conventional bipolar mapping with Advisor™ HD-Grid mapping collects an optimal local EGM from orthogonal bipolar pairs [1 and 2]. On the other hand, novel omnipolar mapping selects an optimal local EGM from omnipolar EGMs calculated from cliques, composed of 3 unipoles and 2 orthogonal bipoles, resulting in EGMs in 360° at each clique [[3], [4], [5]]. Omnipolar EGMs take advantage of both basic unipolar and bipolar signals, providing a local signal like a bipolar electrogram with information on the direction and speed of the wavefront like a unipolar electrogram.

**Fig. 3:**
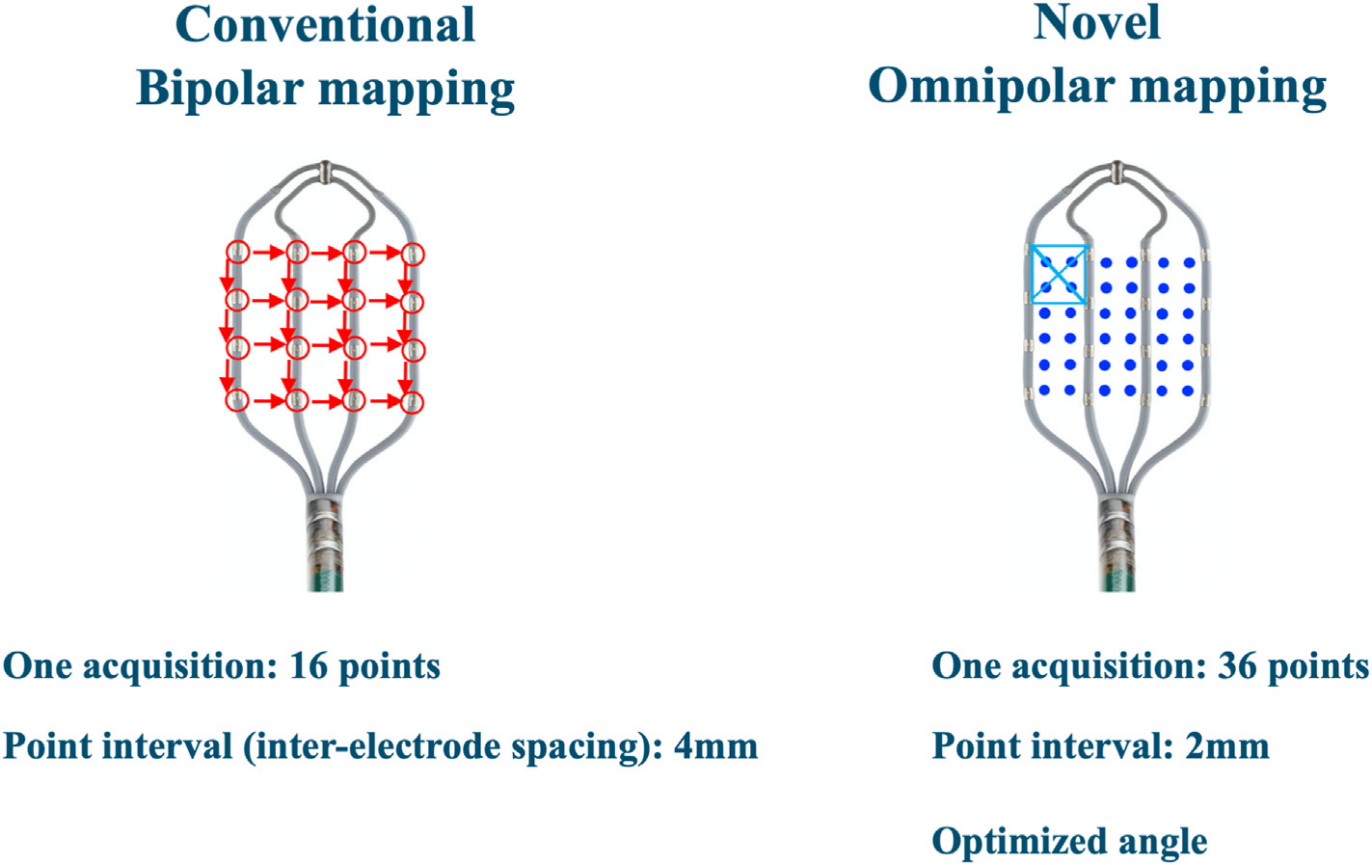
A Difference between the conventional bipolar mapping and novel omnipolar mapping with HD Grid catheter.

As a result, novel omnipolar mapping using Advisor™ HD-Grid mapping catheter with Ensite™ X EP System (Abbott) simultaneously provides three advantages compared to the conventional bipolar mapping (Fig. 3).

First, one acquisition with HD-grid provides 36 acquisition points in novel omnipolar mapping compared to 16 acquisition points in conventional bipolar mapping, simply resulting in an increasing point density. Second, point interval in HD Grid decreases to 2mm with novel omnipolar technology compared to 4mm in conventional bipolar mapping, resulting in increasing mapping resolution. Finally, novel omnipolar technology optimizes the angle between the bipolar EGM measurement and activation direction, automatically eliminating bipolar blindness by selecting EGMs with the largest voltage over 360°, resulting in acquiring the true local EGMs [[3], [4], [5]].

In conclusion, novel omnipolar mapping optimizes the activation angle and increases point density and mapping resolution, allowing the display of the continuous activities at the isthmus in the low voltage area, which may not be possible with conventional bipolar mapping performed with the HD Grid catheter.

## Funding

This work was partially supported by JSPS KAKENHI Grant Number JP20K17074.
